# What’s the Matter?

**Published:** 1877-07-20

**Authors:** 


### WHAT'S THE MATTER?

In an interesting paper upon the subject of vital statistics, in *Sanitarian* the the writer, in alluding to the decline in birth-rate in *New England*, attributes it to a decline in the normal standard of physiological development, by which the law of propagation is impaired, etc. We think it requires no fine-spun theory of this kind to account for the fall-off in births. It is there as in France. The cause is unquestionably to be found in the work of the abortionists, and in methods used to prevent conception. The remote cause is a spirit of rebellion against the order of Nature for the multiplication of the species—a decline in virtue, and an increasing demoralization of the masses.

